# Plasmacytoid variant of bladder cancer defines patients with poor prognosis if treated with cystectomy and adjuvant cisplatin-based chemotherapy

**DOI:** 10.1186/1471-2407-13-71

**Published:** 2013-02-08

**Authors:** Bastian Keck, Sven Wach, Robert Stoehr, Frank Kunath, Simone Bertz, Jan Lehmann, Michael Stöckle, Helge Taubert, Bernd Wullich, Arndt Hartmann

**Affiliations:** 1 Department of Urology, University Erlangen, Krankenhausstraße 12, Erlangen, 91054, Germany; 2 Department of Pathology, University Erlangen, Krankenhausstr 8-10, Erlangen, 91054, Germany; 3 Urology Practice, Prüner Gang, Prüner Gang 15, Kiel, 24103, Germany; 4 Department of Urology, Saarland University, Kirrberger Straße, Homburg/Saar, 66421, Germany

**Keywords:** Chemotherapy, Cystectomy, Micropapillary, Plasmacytoid, Prognosis, Urinary bladder neoplasm

## Abstract

**Background:**

Since the definition of different histologic subtypes of urothelial carcinomas by the World Health Organization (WHO) 2004 classification, description of molecular features and clinical behavior of these variants has gained more attention.

**Methods:**

We reviewed 205 tumor samples of patients with locally advanced bladder cancer mainly treated within the randomized AUO-AB05/95 trial with radical cystectomy and adjuvant cisplatin-based chemotherapy for histologic subtypes. 178 UC, 18 plasmacytoid (PUC) and 9 micropapillary (MPC) carcinomas of the bladder were identified. Kaplan Meier analysis and backward multivariate Cox’s proportional hazards regression analysis were performed to compare overall survival between the three histologic subtypes.

**Results:**

Patients suffering from PUC have the worst clinical outcome regarding overall survival compared to conventional UC and MPC of the bladder that in turn seem have to best clinical outcome (27.4 months, 62.6 months, and 64.2 months, respectively; p=0.013 by Kaplan Meier analysis). Backward multivariate Cox´s proportional hazards regression analysis (adjusted to relevant clinicopathological parameters) showed a hazard ratio of 3.2 (p=0.045) for PUC in contrast to patients suffering from MPC.

**Conclusions:**

Histopathological diagnosis of rare variants of urothelial carcinoma can identify patients with poor prognosis.

## Background

**Figure 1:**
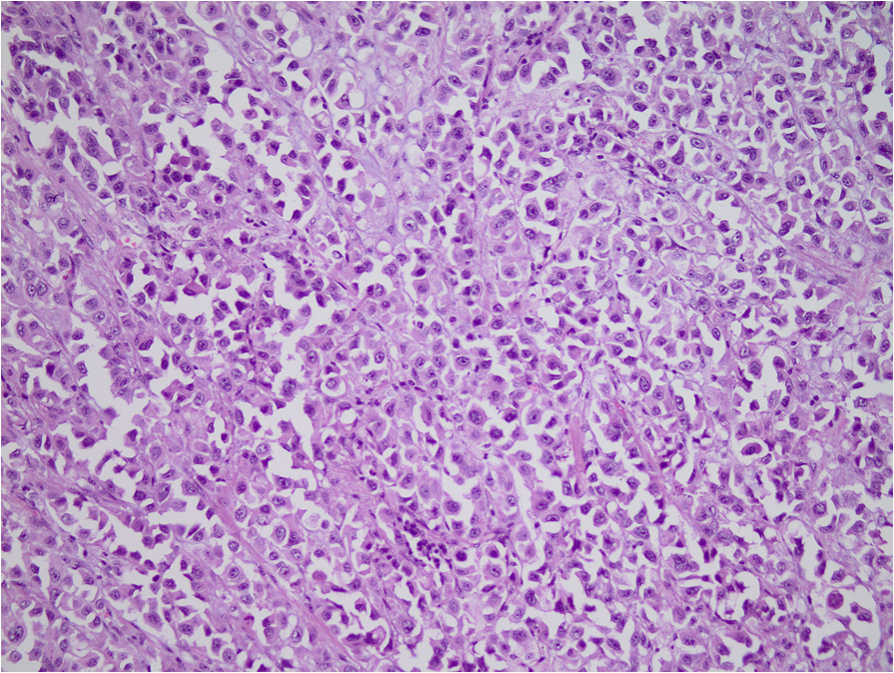
Hematoxylin and eosin staining (200x): Plasmacytoid urothelial carcinoma showing a characteristic single cell growth pattern with eccentrically located nuclei and abundant eosinophilic cytoplasm.

Urothelial carcinoma (UC) is one of the most common malignancies with a total of 110,500 new cases diagnosed per year in Western Europe [1]. The plasmacytoid urothelial carcinoma of the bladder (PUC) and the micropapillary carcinoma of the bladder (MPC) represent two of several rare variants of urothelial carcinoma, which were included in the World Health Organisation (WHO) classification in 2004 [2]. Each of these subtypes accounts for approximately 3-5% of UC in patients with muscle infiltration. Morphologically, PUC presents with a discohesive, single cell growth pattern, with eccentrically located nuclei and an abundant eosinophilic cytoplasm (Figure 1) [3]. PUC is usually diagnosed at an advanced pathological stage and survival appears to be more unfavourable to what has been described for conventional UC [4]. MPC was first described by Amin et al. in 1994 [5] and is characterised by medium sized cells surrounded by an eosinophilic cytoplasm with an irregular distribution of chromatin, frequent mitotic figures and nuclear pleomorphisms [5-8]. Because of this original identification, multiple reports describing the aggressive biologic behaviour of MPC and its unfavourable clinical course, in mainly small cohorts, have been published. Clinical data comparing the different variants of UC and their impact on survival of patients treated with cystectomy and an adjuvant cisplatin-based chemotherapy are still lacking. Thus, we describe for the first time the clinical behaviour of MPC and PUC in comparison to UC in patients treated with cystectomy and adjuvant cisplatin-based chemotherapy within the prospective and randomized trial AUO-AB05/95.

## Methods

**Figure 2:**
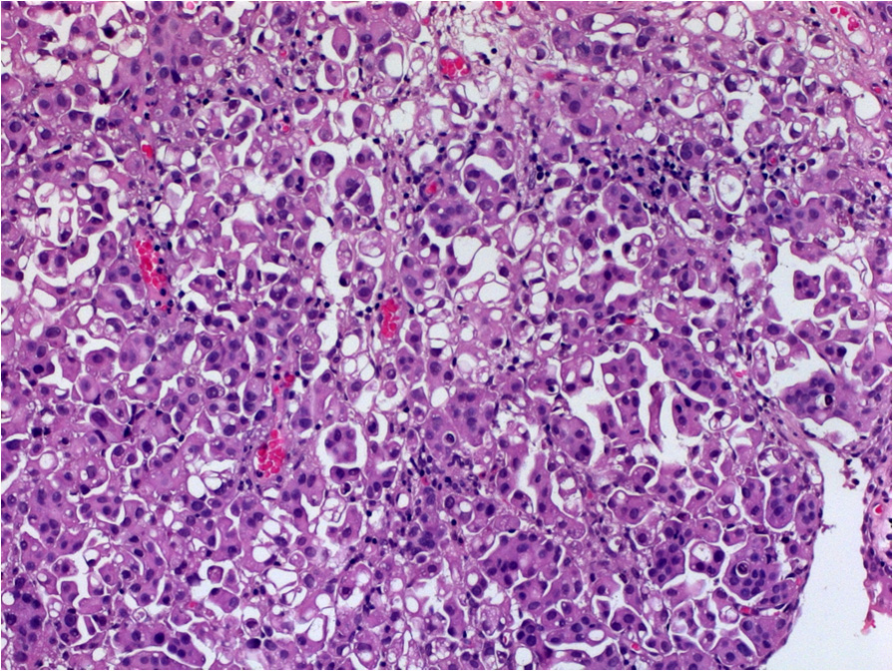
Hematoxylin and eosin staining (200x): Micropapillary carcinoma with medium sized tumor cells and eosinophilic cytoplasm that typically arrange in small nests and show slender, delicate processes, often with a central fibrovascular core.

For this study 221 tissue samples of patients suffering from locally advanced bladder cancer were analysed. Of the 205 patients, 166 tissue samples from the AUO-AB05/95 trial comparing methotrexate, vinblastine, epirubicin, and cisplatin (M-vec) (80 patients) and cisplatin plus methotrexate (Cm) (86 patients) were available. These patients were randomly assigned to the therapy. 39 patients with locally advanced bladder cancer were treated with radical cystectomy and adjuvant chemotherapy with gemcitabine, cisplatin (Gc) according to current guidelines were added to analysis, None of the patients received neoadjuvant chemotherapy. The study protocol of the AUO-AB 05/95 trial was approved by the committee of the “Arbeitsgemeinschaft Urologische Onkologie” of the German Cancer Society and the local ethics committees of the participating centers. All tissue samples were obtained with approval of the ethical committee of the Friedrich-Alexander-University Erlangen-Nuremberg. For this paper histological workup of tissue samples of radical cystectomy from these patients was done retrospectively from large sections by an experienced uropathologist (AH) to identify the histologic subtypes of muscle invasive bladder cancer including determination of the histological type. Tumors were defined as variant histology (PUC and MPC) if they showed at least 50% of the specific histology. Squamous cell carcinomas, nested-type carcinomas and small cell carcinomas were excluded from analysis. Grading was performed according to the WHO classification of 1973 and the one of 2004. The study population and details of the AUO-05/95 trial have been reported previously [9]. In addition to 178 UC cases, 18 PUC (Figure 1) and 9 MPC (Figure 2) cases were identified. A Kaplan Meier analysis and a multivariate Cox’s proportional hazards regression analysis were performed to compare overall survival between the three histologic subtypes. Statistical tests were performed using the IBM SPSS Statistics 19 software. All of the tests were two-sided, and a *P* value <0.05 was regarded as statistically significant (IBM SPSS, Chicago, IL, USA). Correlations between the histological subtypes and the clinicopathological parameters were determined with the Fisher’s exact test but between them and age with the *t*-test. Correlations between overall survival and histological subtypes were studied univariately with a Kaplan Meier analysis (log rank test) and multivariately with a Cox’s regression hazard analysis (adjusted to age, sex, tumor grade, tumor stage, lymph node and metastases status, type of chemotherapy).

## Results

**Figure 3:**
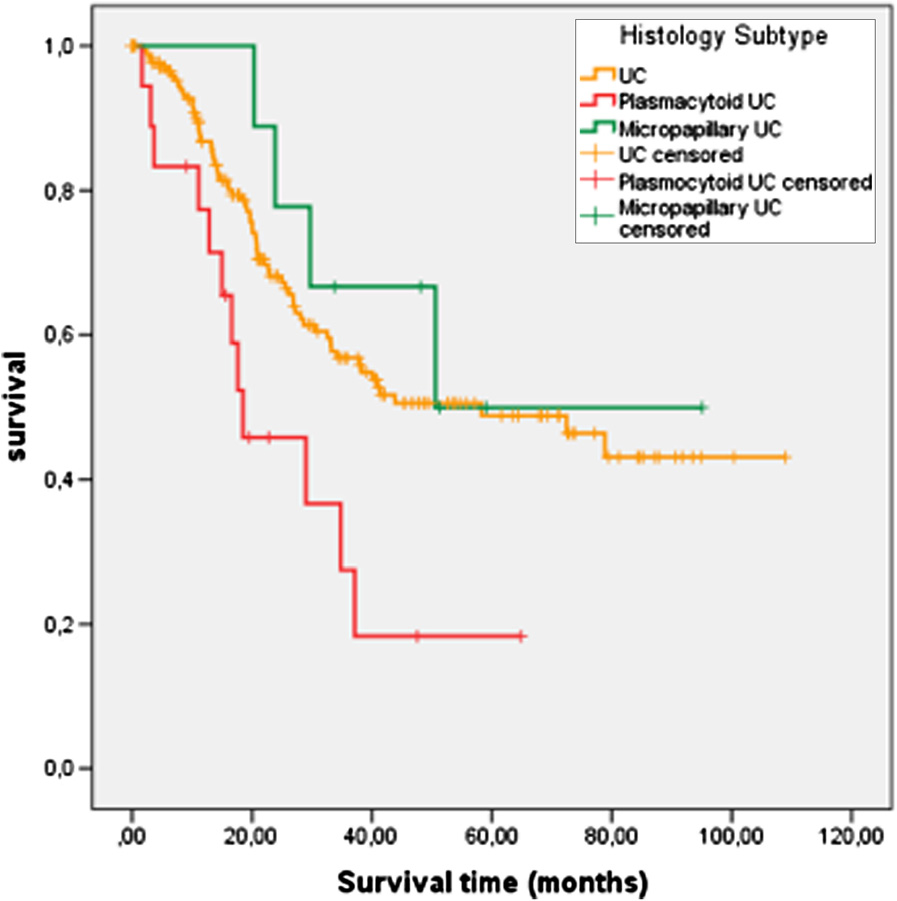
**Kaplan-Meier analysis: Correlation of histology subtype with overall survival.** Patients with a plasmacytoid urothelial cancer (lower curve, N=18) showed with 27.4 months (range: 16.8-37.9) the shortest overall survival, patients with a conventional UC (middle curve, N=178) survived in average 62.6 months (range: 54.8-70.4) whereas patients with a micropapillary urothelial cancer possessed the longest average survival with 64.2 months (range: 41.9-86.4; upper curve N=9). The mean survival was significantly different between patients with plasmacytoid urothelial cancer and those with micropapillary urothelial cancer (P=0.013; log rank test). Censoring of patients (marked with a cross) means mathematically removing a patient from the survival curve at the end of his/her follow-up time.

**Figure 4:**
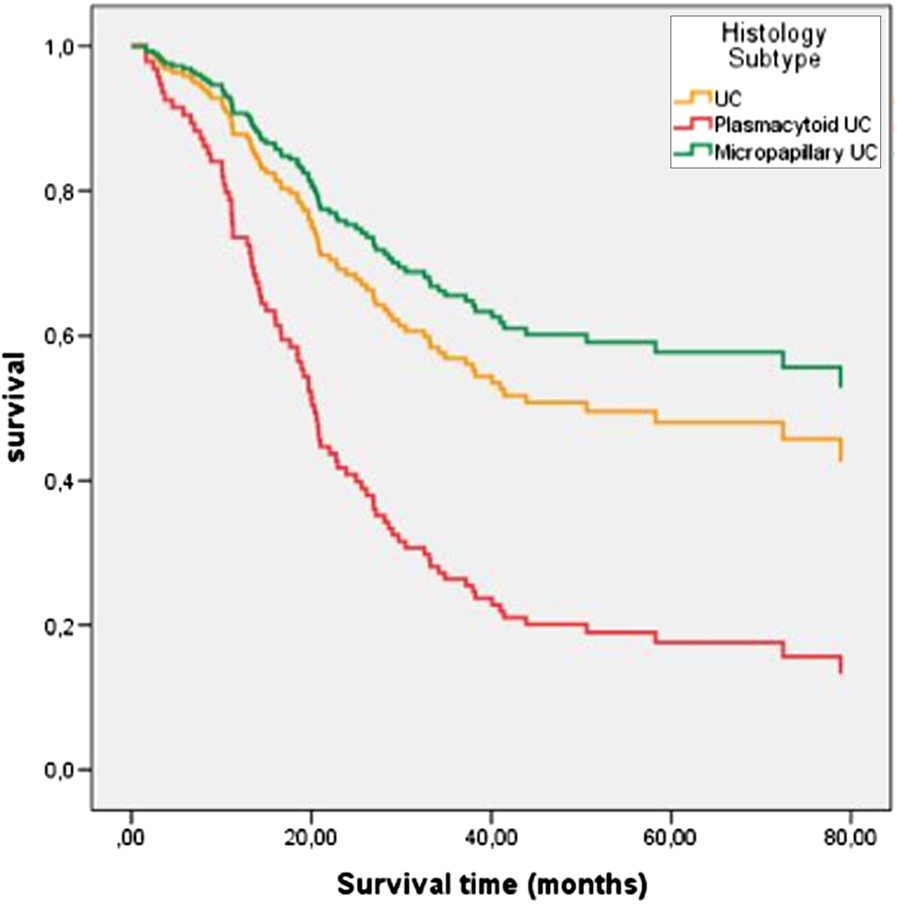
**Multivariate Cox’s regression hazard analysis (adjusted to age, sex, tumor grade, tumor stage, lymph node and metastases status, type of chemotherapy): Correlation of histology subtype with overall survival.** Patients with a plasmacytoid urothelial cancer (lower curve; N=18) have a 3.2-fold (95% CI: 1.0-9.9; P=0.045) increased risk of death while patients with conventional UC (middle curve; N=178) have a 1.3-fold (95% CI: 0.5-3.7; P=0.558) but not significant increased risk of death compared with patients with a micropapillary urothelial cancer (upper curve, N=9).

Clinicopathological parameters of the included patients are shown in Table 1. Although a limited number of patients were included from the MPC and PUC tumor types, they appear to be more frequent in male patients than in female patients (UC: 3.3:1, PUC: 8:1, MPC 9:0). The mean age of the PUC patients seemed to be somewhat lower than that of patients suffering from UC or MPC (57.9, 61.0, and 62.3 years, respectively, p= 0.057). A significant difference for chemotherapy treatment between the different histotypes (P=0.043; Fisher’s exact test; Table 1) is detected. This difference is reasoned by the fact that only UC patients but not MPC or PUC patients have been treated with gemcitabine and cisplatin. However, when we consider only M-vec and Cm treatment regimens no significant difference between the different histotypes is detected (P=0.451; Fisher’s exact test; data not shown). Overall survival of PUC patients was significantly worse than that of patients suffering from UC or MPC (27.4 months with 95% CI: 16.8-37.9 months, 62.6 months with 95% CI: 54.8-70.4 months, and 64.2 months with 95% CI: 41.9-86.4, respectively; p=0.013 using Kaplan Meier analysis; Figure 3). A backward multivariate Cox´ s proportional hazards regression analysis including clinicopathological parameters (age, sex, tumor grade, tumor stage, lymph node and metastases status, type of chemotherapy) showed a hazard ratio of 3.2 (95% CI: 1.0-9.9; p=0.045) for PUC in contrast to patients suffering from MPC (Table 2 and Figure 4). When we analyzed the hazard ratio of the PUC patients compared with the UC patients with the same backward model we still see a 2.4-fold (95% CI: 1.3-4.4; p=0.006) increased risk of death for the PUC patients but no significant difference in risk of death between UC and MPC patients was seen (data not shown). The lymph node status was only in the first step significantly associated with the risk of death but at further backward steps only a trend towards significance was observed (Table 2).

**Table 1:** Comparison of clinicopathological parameters of conventional UC, PUC and MPC urothelial carcinomas treated with radical cystectomy and adjuvant cisplatin based chemotherapy

	**Total**	**UC**	**PUC**	**MPC**	P-Value*
**Age**					0.057**
**Range**	29-75	29-75	46-70	56-70	
**Mean**	60.9	61.0	57.9	62.3	
**Median**	61.7	61.9	59.3	60.8	
**Gender**	205	178	18	9	0.116
**Female**	45 (22%)	43 (24%)	2 (11%)	0 (0%)	
**Male**	160 (78%)	135 (76%)	16 (89%)	9 (100%)	
**pT**	205	178	18	9	0.168
**pT1**	5 (2,5%)	5 (3%)	0 (%)	0 (0%)	
**pT2**	22 (11%)	19 (11%)	3 (17%)	0 (0%)	
**pT3**	142 (69%)	125 (70%)	13 (72%)	4 (45%)	
**pT4**	36 (17.5%)	29 (16%)	2 (11%)	5 (55%)	
**Grade (1973)**	205	178	18	9	0.263
**Grade 2**	22 (11%)	22 (12%)	0 (0%)	0 (0%)	
**Grade 3**	183 (89%)	156 (88%)	18 (100%)	9 (100%)	
**Grade (2004)**					
**Low-grade**	0%	0%	0%	0%	
**High-grade**	100%	100%	100%	100%	
**pN**	205	178	18	9	0.743
**pN0**	90 (44%)	80 (45%)	6 (33%)	4 (44.4%)	
**pN1**	45 (22%)	40 (22.5%)	3 (17%)	2 (22.2%)	
**pN2**	69 (33.5%)	57 (32%)	9 (50%)	3 (33.3%)	
**pN3**	1 (0.5%)	1 (0.5%)	0 (0%)	0 (0%)	
**pM**	205	178	18	9	1.000
**pM0**	204 (99.5%)	177	18 (100%)	9 (100%)	
**pM1**	1 (0.5%)	1	0 (0%)	0 (0%)	
**Chemotherapy**	205	178	18	9	0.043
**Gc**	39	39 (%)	0 (0%)	0 (0%)	
**M-vec**	80	64 (%)	11 (61%)	5 (55%)	
**Cm**	86	75 (%)	7 (39%)	4 (45%)	
**Survival**	205	178	18	9	0.061
**Alive**	120	109	6 (33.3%)	5 (55%)	
**Dead**	85	69	12 (66.6%)	4 (45%)	

Abbreviations: Gc - Gemcitabine, cisplatin, M-vec - Methotrexate, vinblastine, epirubicin and cisplatin, Cm – Cisplatin, methotrexate.

* statistical test: Fisher’s exact test.

** statistical test: *t*-test.

**Table 2:** Backward Cox’s proportional hazards regression analysis

**Variables in the equation**^**b**^									
		**B**	**SE**	**Wald**	**df**	**Significance**	**Exp(B)**	**95.0% Confidence interval for Exp(B)**	
								**lower**	**upper**
Step 1	age	-.151	.232	.422	1	.516	.860	.546	1.356
	gender	-.046	.281	.027	1	.870	.955	.550	1.657
	pT			.432	3	.933			
	pT(1)	.146	.795	.034	1	.854	1.157	.244	5.495
	pT(2)	.047	.464	.010	1	.919	1.048	.422	2.602
	pT(3)	.185	.307	.364	1	.546	1.203	.659	2.196
	Grade	.301	.351	.733	1	.392	1.351	.679	2.690
	pN	-.468	.239	3.842	1	**.050**	.626	.392	1.000
	M			.	0^a^	.			
	Adjuvant Chemo			2.790	2	.248			
	Adjuvant Chemo(1)	-.359	.538	.446	1	.504	.698	.243	2.005
	Adjuvant Chemo(2)	.313	.233	1.806	1	.179	1.368	.866	2.160
	Histo subtype			7.017	2	**.030**			
	Histo subtype(1)	1.095	.588	3.463	1	.063	2.988	.943	9.466
	Histo subtype (2)	.261	.535	.237	1	.626	1.298	.455	3.703
Step 3	age	-.148	.221	.446	1	.504	.863	.559	1.331
	gender	-.078	.275	.081	1	.776	.925	.539	1.585
	Grade	.270	.335	.650	1	.420	1.310	.679	2.528
	pN	-.436	.226	3.737	1	.053	.646	.415	1.006
	Adjuvant Chemo			2.621	2	.270			
	Adjuvant Chemo(1)	-.360	.538	.448	1	.503	.697	.243	2.002
	Adjuvant Chemo(2)	.294	.229	1.642	1	.200	1.342	.856	2.103
	Histo subtype			7.587	2	**.023**			
	Histo subtype(1)	1.150	.581	3.920	1	**.048**	3.157	1.012	9.852
	Histo subtype(2)	.298	.523	.325	1	.569	1.348	.483	3.759
Step 4	age	-.152	.221	.474	1	.491	.859	.558	1.324
	Grade	.270	.336	.649	1	.421	1.310	.679	2.529
	pN	-.437	.226	3.743	1	.053	.646	.415	1.006
	Adjuvant Chemo			2.576	2	.276			
	Adjuvant Chemo(1)	-.375	.535	.490	1	.484	.687	.241	1.963
	Adjuvant Chemo(2)	.285	.227	1.576	1	.209	1.330	.852	2.075
	Histo subtype			7.516	2	**.023**			
	Histo subtype(1)	1.155	.580	3.959	1	**.047**	3.173	1.017	9.895
	Histo subtype(2)	.317	.519	.372	1	.542	1.373	.496	3.797
Step 5	Grade	.292	.333	.766	1	.381	1.339	.697	2.574
	pN	-.420	.224	3.522	1	.061	.657	.423	1.019
	Adjuvant Chemo			2.442	2	.295			
	Adjuvant Chemo(1)	-.358	.535	.449	1	.503	.699	.245	1.993
	Adjuvant Chemo(2)	.278	.226	1.506	1	.220	1.320	.847	2.056
	Histo subtype			7.388	2	.**025**			
	Histo subtype(1)	1.144	.580	3.890	1	**.049**	3.141	1.007	9.792
	Histo subtype (2)	.315	.519	.368	1	.544	1.370	.496	3.787
Step 6	pN	-.434	.223	3.771	1	.052	.648	.418	1.004
	Adjuvant Chemo			2.328	2	.312			
	Adjuvant Chemo(1)	-.399	.532	.561	1	.454	.671	.237	1.905
	Adjuvant Chemo(2)	.256	.224	1.298	1	.255	1.291	.832	2.004
	Histo subtype			6.980	2	**.030**			
	Histo subtype (1)	1.145	.580	3.896	1	**.048**	3.143	1.008	9.800
	Histo subtype(2)	.357	.516	.478	1	.489	1.429	.520	3.928
Step 7	pN	-.404	.222	3.307	1	.069	.667	.432	1.032
	Histo subtype			8.123	2	**.017**			
	Histo subtype (1)	1.162	.580	4.015	1	**.045**	3.197	1.026	9.964
	Histo subtype (2)	.302	.515	.344	1	.558	1.352	.493	3.709

a. Degree of freedom is reduced because of constant or linear dependent covariates.

b. Constant or linear dependent covariate M = 1; Significant values are marked in bold face.

Exp(B) equals relative risk value (RR).

Abbreviations and further information.

Age: was grouped in group1 ≤ 60years and group 2 > 60 years.

Gender: female (reference) and male groups.

pT: tumor stage; pT(1)-tumor stage 1: pT(2) tumor stage 2; pT(3): tumor stage 3; (tumor stage 4 is reference).

Grade: tumor grade: tumor grade 2 and tumor grade 3 (reference).

pN: lymph node status, was grouped in group1=N0 and group2=N≥1 (N1, N2, N3, reference group).

M: metastates status, group1without metastases (M0) and group 2 with metastases (M1), only one patient in group 2 therefore no calculations were performed.

Adjuvant Chemo: Adjuvant Chemotherapy in 3 groups: Adjuvant Chemo(1) is treated with Gc = Gemcitabine, Cisplatin, Adjuvant Chemo(2) is treated with M-vec = Methotrexate, Vinblastine, Epirubicin, Cisplatin and the group treated with CM = Cisplatin, Methotrexate is the reference group.

Histo subtype: Histology subtypes in 3 groups: Histo subtype(1) is the plasmacytoid urothelial carcinoma patients’ group, Histo subtype(2) is the conventional urothelial carcinoma patients’ group and the group with the micropapillary urothelial carcinoma patients is the reference group.

At considering that we do not have for all patients the information for cancer-associated death we calculated their cancer-associated risk of death. In Kaplan Meier analysis we detected mean cancer-associated survival rates for PUC patients of 35.5 months, for UC patients of 67.8 months and for MPC patients of 64.2 months but this is not significant (P=0.22, log rank test). In the multivariate Cox’s regression hazard analysis a 2.0-fold increased risk of tumor-related death for PUC patients compared to MPC patients was found but it was not significant (P=0.26) and an 1.8-fold increased risk of tumor-related death for PUC patients compared to UC patients what was again not significant (P=0.15).

## Discussion

Systemic cisplatin-based chemotherapy is regarded as the therapy of choice in metastatic UC. However, the role of adjuvant or neoadjuvant chemotherapy remains under debate [10,11]. Most urological and oncological guidelines recommend neoadjuvant cisplatin-based chemotherapy as the therapy of choice in locally advanced bladder cancer [12]. Because the description of several rare variants of UC of the bladder in the WHO classification of 2004, descriptions of their clinical course and the molecular features have become more prevalent. However, despite improved knowledge regarding the molecular characteristics of the histologic subtypes of bladder cancer, the impact of these differences on systemic therapies is lacking. Most patients with locally advanced UC are treated without regard to the underlying histology, although it has been reported that tumors with variant histology are associated with a higher risk of progression than conventional high grade UC [13]. As for PUC we could show in the largest series described to date, that on the one hand this subtype of UC accumulates prognostic unfavourable molecular features, like such as the loss of CK20, a high proliferation index and p53 accumulation, and as well as on the other hand exhibits characteristic molecular features, like such as a complete loss of membranous E-cadherin expression [4]. Moreover, our results from this study demonstrate that patients suffering from PUC are of younger age than those suffering from UC or MPC. However, therapeutic strategies with radical cystectomy and cisplatin-based chemotherapy does not seem to be as effective for PUC as it is described for locally advanced UC or MPC, even though the number of patients is limited and may serve as a bias. As it was reported recently, a complete response to adjuvant chemotherapy administering M-VEC and neoadjuvant chemotherapy using gemcitabine and cisplatin may occur at least in a subgroup of PUC -patients [14]. Therefore, chemotherapy in a neoadjuvant or adjuvant setting should be part of the therapeutic considerations. In the present study, we could not confirm these results in our analysis of adjuvant cisplatin-based chemotherapy with a median overall survival of 27.4 months, which is approximately half of that observed in the patients suffering from conventional locally advanced UC (Kaplan-Meier analysis). Thus, PUC tumor biology represents a negative prognostic factor for patients suffering from this histologic variant. Loss of E-cadherin as a sign of epithelial-mesenchymal transition (EMT) and upregulation of transcriptional repressors of E-cadherin may contribute to the aggressiveness of these tumors and a possibly reduced sensitivity to chemotherapeutic agents [4,15,16].

Micropapillary carcinomas have been described in different tumor entities, such as colorectal, breast, lung and others [17-19]. Thus, immunohistochemical panels for discriminating micropapillary tumors of different origins have been reported previously. Within this broad molecular panel, CK20 and uroplakin are the best molecular markers to determine urothelial origin of MPC [19]. In MPC, the initial molecular data explaining their unfavourable clinical course were recently identified, with *HER2/neu* gene amplification, amongst others, as a frequent molecular alteration in this variant [20]. Clinical reports suggest these are markers of biologically aggressive carcinoma with frequent lymphatic vessel invasion in TURB specimens and lymph node metastasis [6,8,21]. Moreover, clinical upstaging to locally advanced diseases occurs in the vast majority of the cases and represents a problem in planning therapeutic strategies [22]. In response, Compérat et al. highlighted the importance of adequate tumor sampling, including analysis of the detrusor muscle, to avoid possible upstaging. Moreover, they state that due to the associated aggressive behaviour, the proportion of micropapillary differentiation should be reported in all cases, even if it represents less than 10% of the specimen, as it has prognostic relevance [22]. Additionally, inter-observer reproducibility of the diagnosis of MPC is low [23], which may lead to treatment delays or the use of inappropriate therapeutic strategies adversely affecting patients’ survival. Therefore, pathologists should be aware of the histologic subtypes on diagnosis. Furthermore, urologists or oncologists should take this information into account when planning surgical or chemotherapeutic treatment options. Supporting the recommendations of Compérat and coworkers Kamat et al. postulated that even papillary and non-invasive MPC should be treated by radical cystectomy to prevent progression and systemic disease [22,24]. In their analysis they demonstrated that neoadjuvant cisplatin-based chemotherapy did not result in an improved 5-year overall survival and that intravesical immunotherapy using BCG was not effective in this histologic variant [24]. Most studies on MPC describe poor disease-specific survival following adjuvant chemotherapy [8,20,25]. However, our data are in contrast to the experiences reported previously. We demonstrated that the survival rates were comparable for MPC and UC if treated with radical cystectomy and adjuvant chemotherapy. These contradictory results may be explained by the prospective randomized nature in which the patients were recruited and included only upon the ability to compare UC, MPC and PUC within a single trial. Although the relatively low number of patients suffering from MPC or PUC may limit the value of our study, it provides important information regarding their clinical course and the aggressive biology of the tumor subtypes. A possible limitation of our study might be the interobserver variability in defining histological subtypes as there is still no consensus on the optimal cut off value of variant histology in the specimen to define PUC or MPC. Another limitation of our results is the measurement of overall survival in our series as this could be affected by several variables besides tumor characteristics. However, on the other hand chemotherapy can have effects on comorbidity and therefore finally affect overall survival what is of relevance for the patients. Awareness of these different bladder cancer variants appears to be crucial when analyzing the molecular characteristics of advanced bladder cancers and when tailoring personalized therapeutic procedures in the future.

## Conclusion

The specific tumor histology gives important prognostic information of patients suffering from locally advanced bladder cancer treated by radical cystectomy and adjuvant chemotherapy. Our results implicate that determining the exact pathological diagnosis, including the description of histologic subtypes of bladder cancers according to the WHO classification of 2004, are important. As UC, PUC and MPC are associated with a different clinical course if treated with cystectomy and adjuvant cisplatin-based chemotherapy prospective multicenter studies, comparing the different histologic variants of bladder cancer and their molecular features are necessary to tailor therapeutic strategies in the future. Furthermore, a joint study including the collection of rare bladder cancers is strongly warranted with a goal of enforcing additional molecular studies for the identification of potential prognostic and therapeutic targets.

## Abbreviations

PUC: Plasmacytoid Urothelial Carcinoma; MPC: Micropapillary Urothelial Carcinoma; UC: Urothelial Carcinoma; M-vec: Methotrexate, Vinblastine, Epirubicin, And Cisplatin; Cm: Cisplatin, Methotrexate; Gc: Gemcitabine, cisplatin; EMT: Epithelial-Mesenchymal Transition.

## Competing interests

The authors declare that they have no competing interest.

## Authors’ contributions

BK designed the study, interpreted the data and drafted the manuscript. BW revised the manuscript for important intellectual content and interpreted the data. JL and MS were involved in the data aquision and interpretation, and revised the manuscript for important intellectual content. AH, did the histological review of the samples, reviewed the manuscript and interpreted the data. RS, FK, and SB were involved in revising the manuscript for important intellectual content as well as data interpretation. SW and HT were involved in drafting the manuscript and did the statistical analysis. All authors read, gave comments, and approved the final version of the manuscript.

## Note added in proof

During the revision process Dayyani et al. published in accordance with our findings that PUC is a very aggressive variant of bladder cancer with overall poor outcomes. In addition they report that despite pathologic downstaging of patients treated with neo-adjuvant chemotherapy, relapses commonly occurred and no difference in survival between patients treated with neo-adjuvant chemotherapy compared to initial surgery was observed.

## Pre-publication history

The pre-publication history for this paper can be accessed here:

http://www.biomedcentral.com/1471-2407/13/71/prepub
